# The anatomy of a jackstone: a novel morphometric analysis of a rare urinary calculus

**DOI:** 10.1007/s00240-026-01968-5

**Published:** 2026-03-17

**Authors:** Daniel Mashiach, Sunjum Singh, Marriam Anjum, Alesh Polivka, Rasha Alnajjar, LeeAnn Wang, Patrick Lee, Hendrik Szurmant, Ellen Fricano

**Affiliations:** College of Osteopathic Medicine of the Pacific, Western University of Health Sciences, 309 E. Second St, Pomona, CA 91776-1854 USA

**Keywords:** Cystolithiasis, Calculi, Computed tomography (CT), Bladder, Prostate, Urinary stasis

## Abstract

Jackstone calculi are rare urinary stones characterized by multiple spiculated arms, yet the mechanisms underlying their distinctive morphology remain incompletely understood, in part due to the challenge of quantifying microstructural growth patterns along their complex three-dimensional geometry. In this study, we applied a non-destructive micro-computed tomography morphometric method to analyze a jackstone retrieved from the bladder of a human donor. This approach enabled measurement of incremental layering along individual arms and branches. We hypothesized that distal arm regions would show evidence of more rapid remodeling than proximal regions. Consistent with this hypothesis, six of seven arms and four of six branches demonstrated greater layer number and decreased layer thickness toward their distal tips, supporting an abrasion-mediated remodeling model of jackstone growth. The donor also exhibited marked prostate enlargement and bladder wall hypertrophy, suggesting chronic urinary stasis as the physiological setting that permitted stone retention and arm development. These findings refine existing conceptual models by indicating that abrasion influences layer remodeling frequency rather than increasing protein layer thickness. The imaging and analytic workflow described here provides a reproducible framework for future structural, biochemical, and microbial investigations into jackstone pathogenesis.

**Supplementary Information:**

The online version contains supplementary material available at 10.1007/s00240-026-01968-5.

## Introduction

Jackstone calculi are rare urinary stones that resemble six-pointed toy jacks due to their dense core and surrounding spike-like protrusions. Fewer than twenty confirmed case reports have been reported in the literature [1–19], most commonly occurring in older males, with average ages ranging from 68 to 73-years-old [2, 20]. Jackstones are typically composed of calcium oxalate dihydrate or monohydrate [3, 4], and their loose crystalline structure allows them to be degraded by lithotripsy. Although the exact process of jackstone formation is still unknown, one hypothesis is urine stasis secondary to bladder outlet obstruction [2, 21, 22]. Repetitive abrasion of the stone arms against the bladder wall may also contribute to the formation of a spiculated contour [4].

The complex three-dimensional geometry of jackstones poses significant methodological challenges in analyzing its morphology. The arms and their subsidiary branches curve in multiple planes, making traditional histologic sectioning insufficient to capture microstructural layering throughout the full extent of the stone. Additionally, the alternating radiopaque (mineralized) and radiolucent (proteinaceous) bands observed in jackstones require high-resolution imaging to be measured accurately along curved growth axes. Prior imaging studies have described the presence of alternating layers and hypothesized that the protein-rich core may preferentially recruit more protein than calcium oxalate, allowing the jackstone arms to grow at a faster rate than the base [4], however no reproducible method has been established for quantifying microstructural growth patterns along individual arms.

This paper aims to address this methodological gap and expand upon anatomical observations of jackstone calculi, uniquely utilizing a stone identified and extracted from a human donor, as the first study on a jackstone known to have been found in this manner, to the authors’ knowledge. Using non-destructive CT imaging of the jackstone, we analyzed the morphology and microstructure of this jackstone using a novel morphometric approach.

The primary goal of this study was to test the feasibility of a reproducible micro-CT–based method for quantifying jackstone arm and branch microstructure. The secondary goal was to evaluate whether observed layering patterns support an abrasion-driven remodeling mechanism of stone growth. Based on previous research [4], it was hypothesized that the number of growth layers would be greater toward the distal tips of arms and branches, and individual layer thickness would be thinner at the tips compared to the proximal base.

## Methods

### Gross measurements

A jackstone calculus was retrieved from the bladder of an 88-year-old male cadaveric donor with a documented medical history of coronary artery disease, cardiomyopathy, and hypertension. The dimensions of the jackstone were measured using an electronic caliper. An electronic caliper was also employed to measure the length and width of both kidneys, the right ureter (the left ureter was obliterated during dissection), the bladder, and the prostate.

The prostate was sectioned in the mid-sagittal plane, and its superior-inferior length and medial-lateral width were measured. Due to the curved path of the prostatic urethra, ImageJ was used to measure the accurate length using photographic images of the prostatic urethra that were taken with a standard ruler in the frame. The masses of the bladder and prostate were measured by excising the organs from the donor and weighing them on a digital scale individually and together.

### Micro-CT measurements

**Fig. 1:**
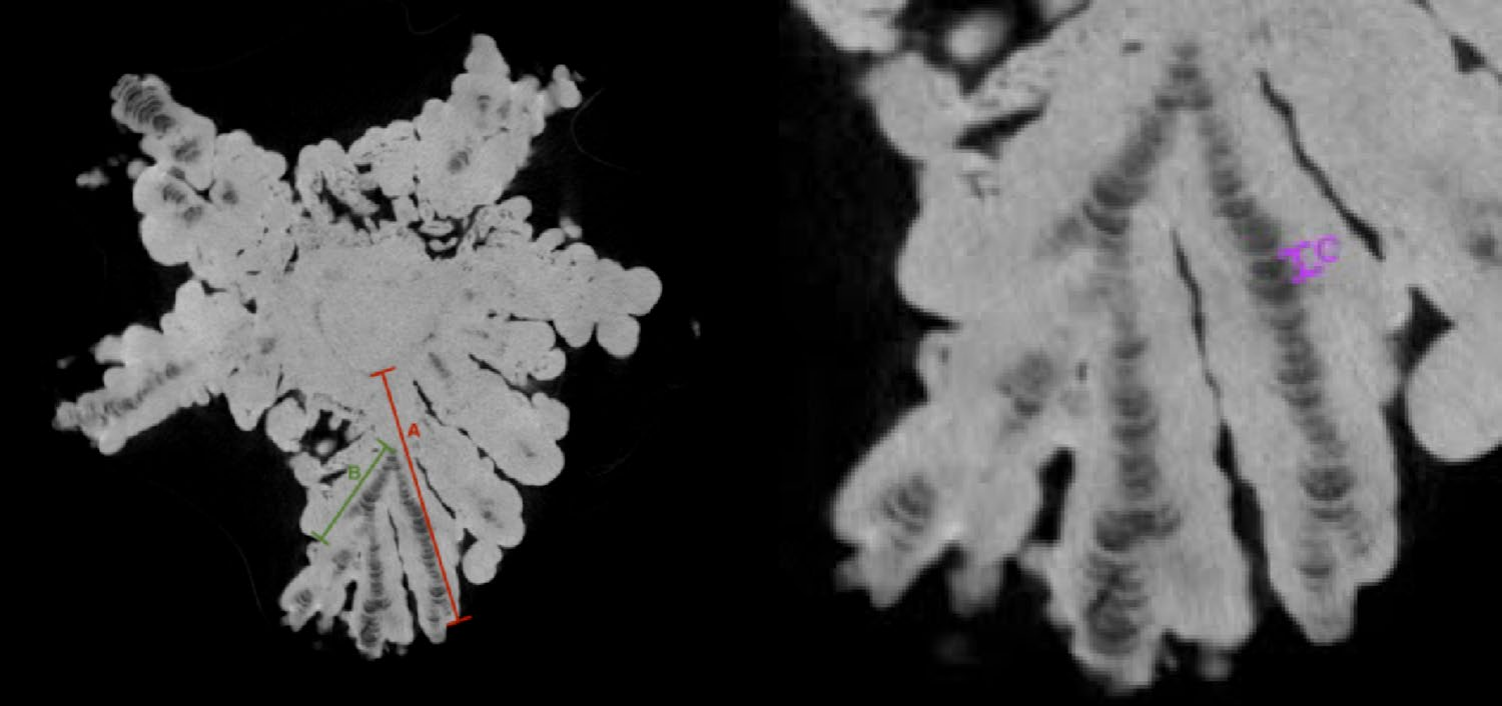
Jackstone segmentation using micro-CT. Label A, in red, is an example of a jackstone arm. Label B, in green, is an example of a branch from arm A. Label C, in purple, is a layer of arm A, which is composed of one radiopaque and one radiolucent layer

Imaging of the jackstone was performed with a SkyScan 1275 High-Speed Micro-CT Scanner using an aluminum filter (voxel size: 19.5 μm, 90 KV, 125 Amp). Image segmentation and visualization were performed in Dragonfly [23]. The total length of arms as well as the proximo-distal lengths of each layer were measured. Each radiopaque and radiolucent increment was measured together as one layer (Fig. 1).

We employed an approach previously used to measure tooth cementum mineralization [24] to characterize the mineralization of the jackstone. To assess spatial trends in microstructure, each arm and branch was divided into proximal (base) and distal (tip) halves. Arms were defined as protrusions originating from the jackstone base, whereas branches were defined as protrusions originating from the jackstone arms (Fig. 1).

### Composition analysis

Stone composition was determined using standard crystallographic analysis. The jackstone was sectioned, and the central core was crushed using a sterile mortar and pestle to obtain a representative sample. The material was submitted to a commercial reference laboratory (Uro-Lithiasis Laboratory Inc.) for optical crystallographic composition analysis. The laboratory performed polarized light microscopy and crystal morphology assessment according to routine clinical stone-analysis protocols.

### Statistical analysis

The relationship between the length and distance of the layers from the base was analyzed using a regression analysis. A Wilcoxon signed-rank test for paired samples was utilized to compare arm versus branch layer thickness with a significance level set at *p* = 0.05. Statistical analysis was performed in SPSS [25].

## Results

### Gross measurements

The left hemisected bladder wall thickness measurements revealed the following: at the fundus (highest point) of the bladder, the thickness of the wall was 16.0 mm, the midline thickness of the anterior wall was 8.50 mm, and the midline posterior wall, where the jackstone impression was noted, was 13.4 mm.

**Fig. 2:**
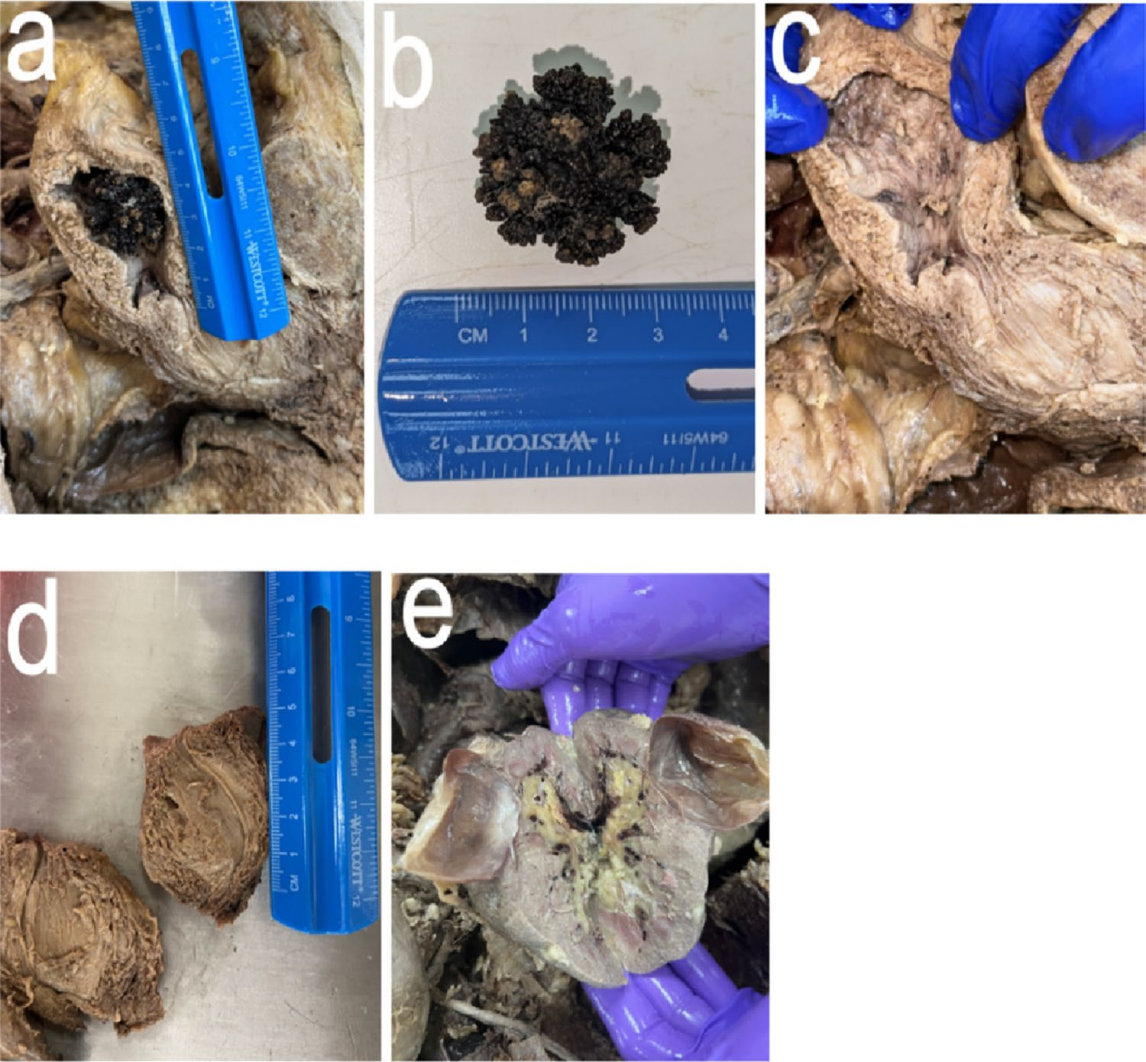
Gross examination of dissected bladder with jackstone located at posterior bladder wall. (**a**) Hemisected bladder with Jackstone, (**b**) Jackstone measuring around 3 cm x 3 cm, (**c**) Hemisected bladder showing jackstone impression at fundus of bladder, (**d**) Hemisected prostate, and (**e**) An external drained cyst located in the left kidney

At the internal urethra, the anterior wall of the hemisected bladder measured 13.7 mm, and the posterior wall measured 19.7 mm (Fig. 2). The height of the internal chamber of the bladder, from the internal urethral sphincter to the fundus, was 80.9 mm, with a transverse diameter of approximately 37.7 mm. The right side of the hemisected prostate measured 52.2 mm in superior-inferior and 43.6 mm in medial-lateral. The length of the right ureter from the right renal hilum to the vesicoureteric junction was 205 mm.

When flattened and empty, the width of the ureters varied along their length. At the upper right portion near the renal pelvis, the right ureter diameter measured 4.90 mm, one-quarter down from that point it increased to 7.03 mm, three-quarters down, it measured 3.60 mm, and at the base of the ureter where it enters the bladder, it was 5.60 mm in width. The widest part of the right ureter, located approximately 116 mm from the renal pelvis, measured 8.80 mm, with a luminal diameter of 5 mm as measured using a conical inner diameter gauge. The left ureter was cut, so its length was not recorded. Its diameter did not vary significantly along its length, and the luminal diameter was measured as 3 mm using a conical inner diameter gauge. Additionally, a single, large, drained cyst in the left kidney was observed with an internal diameter of 45.8 mm. The bladder alone weighed 73.2 g, while the prostate alone weighed 90.7 g. Further dissection of this donor also revealed a significant collection of gallstones.

### microCT measurements

The thickness and number of layers were measured for seven arms and six branches. The mean length of an arm was 9.34 mm, whereas the mean length of a branch was 2.92 mm. Six of the seven arms had more layers towards the tips, with an average difference of 4.14 additional layers closer to the tip. Four of the six branches had more layers towards the tips, with an average difference of 1.33 layers between the two halves (Table 1).

**Table 1:** Number of layers closer to base vs. tips, in arms and branches

	Length (mm)	Base to Mid	Mid to Tip	Difference	Mean difference	Mean Length (mm)
*Arms*						
Arm 1	11.54	16	23	7		
Arm 2	7.28	12	18	6		
Arm 3	8.86	20	23	3		
Arm 4	5.22	6	10	4		
Arm 5	11	27	23	-4		
Arm 6	10.93	12	19	7		
Arm 7	10.55	18	24	6	4.14	9.34
*Branches*						
Branch 1.1	2.25	5	8	3		
Branch 1.2	5.73	11	15	4		
Branch 3.1	2.06	7	6	-1		
Branch 4.1	2.01	3	5	2		
Branch 6.1	2.94	6	5	-1		
Branch 7.1	2.51	5	6	1	1.33	2.92

**Fig. 3:**
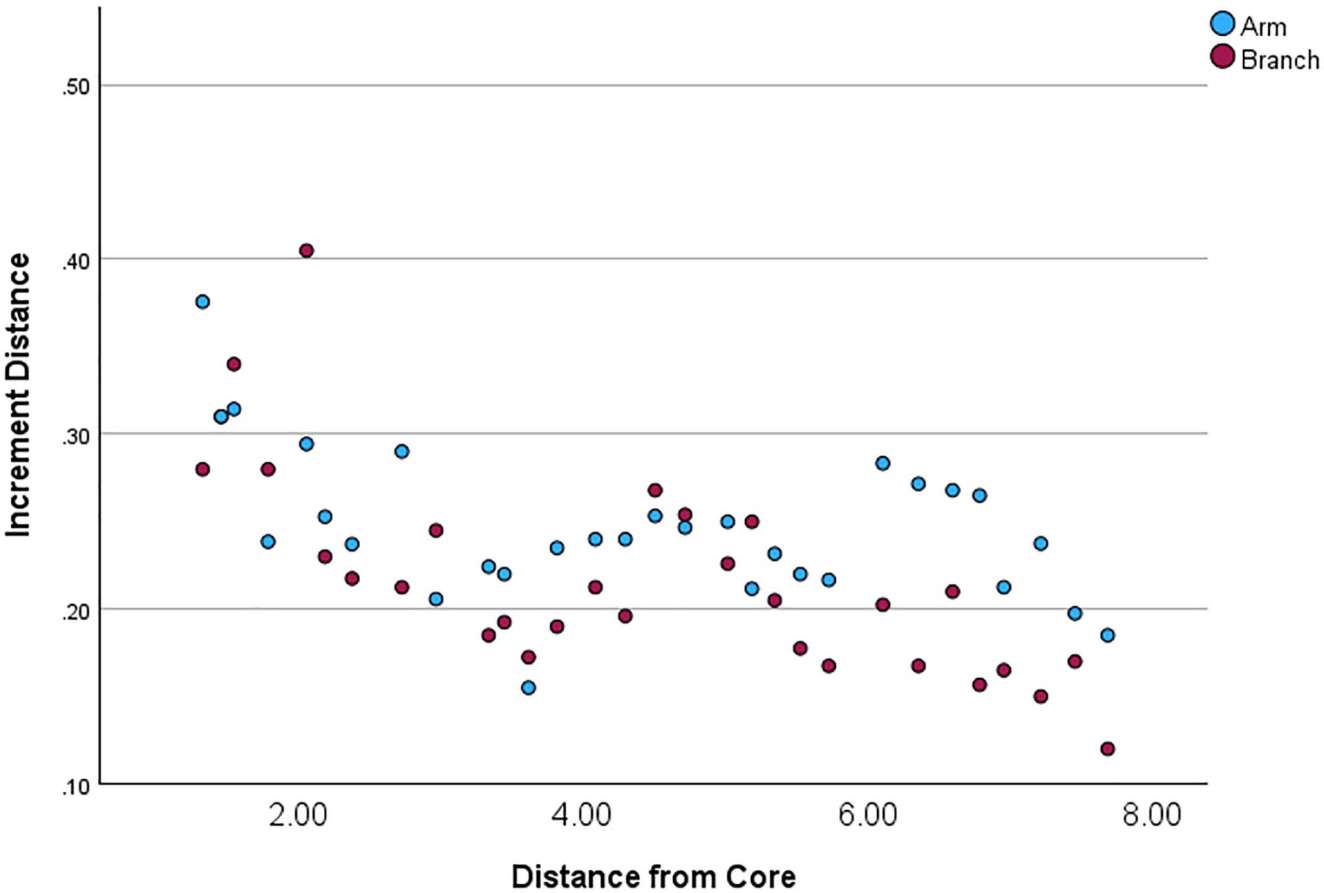
Jackstone layer thickness plotted by distance from the base. Blue points represent arms and red points represent branches

**Fig. 4:**
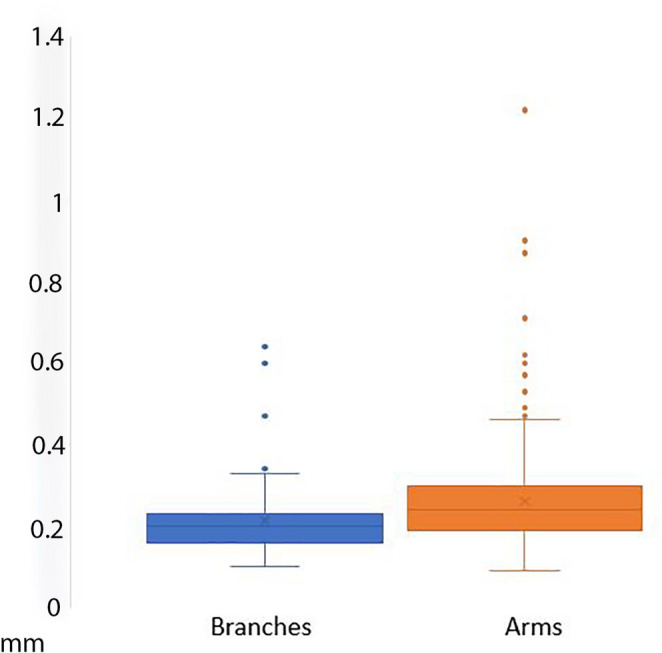
Box plot of arm and branch growth. The blue column represents branch thickness and the orange column represents the arm thickness of the jackstone

In the measured jackstone arms, an inverse relationship between the thickness of layers and distance from the base was noted (Fig. 3). A statistically significant trend (*p* < 0.001) of decreasing thickness was observed as measurements were recorded further from the base and closer to the tip of each arm (Table 2). Additionally, branches showed statistically significant (*p* = 0.002) thinner layers than arms (Fig. 4; Supplementary Tables 1–3).

**Table 2:** Statistical correlations

		Distance from Base	Average Branch Increment	Average Arm Increment
Distance from Base	Pearson Correlation	1	-0.724**	-0.482**
	Sig (2-tailed)		< 0.001	< 0.001
	N	50	30	50
Average Branch Increment	Pearson Correlation	-0.724**	1	0.590**
	Sig (2-tailed)	< 0.001		< 0.001
	N	30	30	30
Average Arm Increment	Pearson Correlation	-0.482**	0.590**	1
	Sig (2-tailed)	< 0.001	< 0.001	
	N	50	30	50

**Correlation is significant at the 0.01 level (2-tailed)

### Composition analysis

Jackstones described in the literature are typically composed of calcium oxalate monohydrate or calcium oxalate dihydrate (3,4). To confirm that the donor-derived jackstone examined in this study is representative of urinary jackstones in general, we crushed the core of the stone with a mortar and pestle and submitted the material for composition analysis to a commercial laboratory (Uro-Lithiasis Laboratory Inc.). Optical crystallographic analysis revealed a composition of 97% calcium oxalate monohydrate, 2% calcium oxalate dihydrate, and 1% calcium phosphate (apatite). These results support the conclusion that our findings can be generalized to bladder jackstone formation.

## Discussion

This study demonstrates a new micro-CT–based morphometric method for quantifying layering patterns in jackstone calculi, providing a reproducible framework for evaluating jackstone growth mechanisms. Using this method, we show that the jackstone arms exhibit more numerous and thinner layers toward their distal tips, supporting a model of abrasion-mediated remodeling during stone growth.

In addition to the microstructural findings, the anatomical context of the donor provides important physiological insight into jackstone formation. The markedly enlarged prostate and pronounced bladder wall hypertrophy are anatomical changes commonly associated with chronic bladder outlet obstruction and urinary stasis, a well-established risk factor for urinary stone formation [26–28]. For reference, the average bladder weight in adult males is approximately 25–30 g [29], whereas the donor’s bladder weighed 73.2 g. Similarly, the donor’s prostate measured 90.7 g, substantially exceeding the typical weight of 20–30 g in normal prostates and 30–50 g in benign prostatic hyperplasia [30].

Chronic obstruction increases post-void residual volume and reduces urinary flow, conditions that favor crystal nucleation, retention, and incremental stone growth [26]. As the bladder compensates, muscular hypertrophy and inflammation develop, contributing further to urinary stasis. In this donor, the combination of bladder wall hypertrophy and significant prostate enlargement supports the interpretation that persistent urinary retention created a biochemical and mechanical environment conducive to jackstone formation [27, 28].

From this clinical context, we then turn to the microstructural patterning revealed by micro-CT imaging, which allows us to evaluate the mechanism by which the arms remodel and extend over time. Our method provides a means by which microstructural patterns may be evaluated and further provides anecdotal support for the hypothesis that the distinctive morphology of jackstone calculi results from the accelerated radial growth of their arms compared to the base, due to physical abrasion between the stone and the bladder wall [4]. Specifically, the distal regions of the arms contact the bladder wall, leading to the stripping of proteins and/or minerals and subsequent exposure of the arm tips. This exposure likely increases remodeling at these sites, promoting further growth, and as the jackstone arms grow, abrasion becomes more frequent, leading to a redundant cycle. The repetitive nature of this abrasion and binding cycle results in the formation of progressively thinner concentric layers over time, as supported in our findings.

Our method provides a technique that would allow the abrasion-driven growth model to be tested, elucidating the unique morphology of jackstone calculi. Canela et al. (2022) suggested that post-abrasion, the exposed protein layer attracts additional proteins, thereby extending the protein layer [4]. If this were the case, an increase in layer thickness, particularly of the radiolucent protein layer, would be anticipated. However, our observations do not corroborate this, as we did not detect evidence of protein-to-protein increased attraction leading to thicker protein layers. Instead, our results indicate that while abrasion may facilitate preferential binding, it does not necessarily result in increased protein layer thickness. This suggests a modified understanding of the growth mechanism, where abrasion influences the pattern of layer remodeling without significantly altering protein layer thickness. These insights contribute to a more nuanced comprehension of the formation and development of jackstone calculi.

This study enhances current knowledge of bladder stone formation mechanisms and, with future research building upon it, may help guide screenings, diagnostics, and clinical treatments [31]. With insight into the link between urinary stasis and stone formation, clinicians can implement earlier interventions in high-risk patients with urinary retention. Additionally, a potential therapeutic approach could focus on reducing the abrasion a bladder stone has with the wall to minimize the remodeling and growth.

There were a few inherent limitations to this work. This study examined a single specimen, limiting generalizability, however the purpose of this work was to establish a methodological framework for future sample-comparative studies. The relative rarity of the condition makes the small sample size nearly unavoidable. Another limitation is the postmortem changes that may have occurred in the donor, including tissue breakdown and loss of muscle tone. However, while the organ weights were taken from a donor patient, one study compared the bladder weights of 10 male donor cadavers, with a mean age of 67 ± 21 years, to ultrasound-estimated bladder weights in living patients and found no significant difference between the bladder weights of cadaveric patients and those of the living cohort [29].

Given the apparent association between urinary stasis and jackstone formation, future studies should explore the potential microbial contributions to this process, as culturing and sequencing jackstone specimens may uncover distinct microbial signatures or biofilm communities associated with their development. With the urinary microbiome now recognized as an integral component of urologic health [32], it is plausible that prolonged urinary stasis may foster colonization by specific bacterial taxa that promote or modulate jackstone formation. This hypothesis is supported by emerging evidence linking particular microbial species to the formation of other stone subtypes [33], warranting further investigation into the microbiological underpinnings of jackstone pathogenesis.

## Conclusion

This study establishes a reproducible micro-CT–based morphometric approach for evaluating microstructural growth patterns in jackstone calculi. Using this method, we demonstrated that the arms exhibit more numerous and thinner incremental layers toward their distal tips, a pattern consistent with abrasion-mediated remodeling at sites of repeated contact with the bladder wall. The anatomical findings in the donor, specifically, pronounced prostate enlargement and bladder wall hypertrophy, further support a clinical setting of chronic urinary stasis, which likely provided the physiological conditions necessary for stone retention and progressive arm development.

Together, these structural and clinical observations refine current models of jackstone formation by suggesting that abrasion influences layer remodeling frequency rather than increasing protein layer thickness. The imaging and analytic workflow described here provides a framework for future comparative studies, including biochemical and microbial analyses, to further clarify the mineralization dynamics and potential biological contributors to jackstone pathogenesis. Continued investigation using larger sample cohorts will be essential for defining the variability of growth patterns across jackstone phenotypes and for informing targeted strategies to mitigate stone progression in patients at risk.

## Supplementary Information

Below is the link to the electronic supplementary material.

Supplementary Material 1

## Data Availability

The data that supports the findings of this study are available from the corresponding author upon reasonable request.
