# Supplementary material for: The anatomy of a jackstone: a novel morphometric analysis of a rare urinary calculus
Source: Urolithiasis. 2026 Mar 17;54(1):66. doi: 10.1007/s00240-026-01968-5 (PMC12995973; doi:10.1007/s00240-026-01968-5)
Supplement: Supplementary file 1 — Supplementary Material 1 [file 240_2026_1968_MOESM1_ESM.pdf]

The Anatomy of a Jackstone:
A Novel Morphometric Analysis of a Rare Urinary Calculus

Daniel Mashiach[1], Sunjum Singh[1], Marriam Anjum[1], Alesh Polivka[1], Rasha Alnajar[1], LeeAnn Wang[1], Patrick Lee[1], Hendrik Szurmant[1], and Ellen Fricano[1]*

[1]College of Osteopathic Medicine, Western University of Health Sciences, Pomona, CA

*Corresponding author:
Ellen Fricano, PhD
909-469-5219
efricano@westernu.edu
ORCID ID: 0000-0002-8258-9781
309 E. Second St.
Pomona, CA 91776-1854

Supplementary Material: Statistical analysis of jackstone arm and branch growth

Supplementary Table 1: Descriptive statistics of arm and branch growth.

	N (sample size)	Mean	Std. Deviation	Minimum	Maximum	25th percentile	50th percentile	75th percentile
Branches	99	0.2127	0.08578	0.10	0.64	0.1600	0.2000	0.2300
Arms	288	0.2612	0.12233	0.09	1.22	0.1900	0.2400	0.3000

Supplementary Table 2: Wilcoxon Signed Ranks Test.

Arms - Branches		N	Mean Rank	Sum of Ranks
	Negative Ranks	35 (arms < branches)	42.44	1485.50
	Positive Ranks	21 (arms > branches)	51.98	3170.50
	Ties	3 (arms = branches)		
	Total	99		

Supplementary Table 3: Test statistics.

	Arms - branches
Z	-3.080 (based on negative ranks)
Asymp. Sig (2-tailed)	0.002
